# Gene Expression Analysis of Environmental Temperature and High-Fat Diet-Induced Changes in Mouse Supraclavicular Brown Adipose Tissue

**DOI:** 10.3390/cells10061370

**Published:** 2021-06-02

**Authors:** Yufeng Shi, Honglei Zhai, Sharon John, Yi-Ting Shen, Yali Ran, Giang Hoang, Miao-Hsueh Chen

**Affiliations:** USDA/ARS, Children’s Nutrition Research Center, Department of Pediatrics, Baylor College of Medicine, Houston, TX 77030, USA; yfshi89@gmail.com (Y.S.); Honglei.Zhai@bcm.edu (H.Z.); sharonj2198@gmail.com (S.J.); Yi-Ting.Shen@bcm.edu (Y.-T.S.); Yali.Ran@bcm.edu (Y.R.); vangianghoang94@gmail.com (G.H.)

**Keywords:** adipose tissue, supraclavicular brown adipose tissue, thermogenesis, thermoneutrality, high-fat diet

## Abstract

Obesity, a dysregulation of adipose tissue, is a major health risk factor associated with many diseases. Brown adipose tissue (BAT)-mediated thermogenesis can potentially regulate energy expenditure, making it an attractive therapeutic target to combat obesity. Here, we characterize the effects of cold exposure, thermoneutrality, and high-fat diet (HFD) feeding on mouse supraclavicular BAT (scBAT) morphology and BAT-associated gene expression compared to other adipose depots, including the interscapular BAT (iBAT). scBAT was as sensitive to cold induced thermogenesis as iBAT and showed reduced thermogenic effect under thermoneutrality. While both scBAT and iBAT are sensitive to cold, the expression of genes involved in nutrient processing is different. The scBAT also showed less depot weight gain and more single-lipid adipocytes, while the expression of BAT thermogenic genes, such as *Ucp1*, remained similar or increased more under our HFD feeding regime at ambient and thermoneutral temperatures than iBAT. Together, these findings show that, in addition to its anatomical resemblance to human scBAT, mouse scBAT possesses thermogenic features distinct from those of other adipose depots. Lastly, this study also characterizes a previously unknown mouse deep neck BAT (dnBAT) depot that exhibits similar thermogenic characteristics as scBAT under cold exposure and thermoneutrality.

## 1. Introduction

Obesity prevalence has increased in pandemic dimensions over the past 50 years [1]. It represents a major health challenge as it substantially increases the risk of cardiovascular disease, type 2 diabetes, hypertension, and several forms of cancers, thereby contributing to a decline in both quality of life and life expectancy [2,3]. It is estimated that over 10% of the world population is obese and projected that 1 in 2 adults will become obese in United States by 2030 [4]. Thus, reducing the prevalence of obesity is critical for human health.

Obesity occurs when energy intake exceeds energy expenditure, and adipose tissue plays a major role in regulating energy balance and metabolism [5]. There are two major types of adipose tissues in mammals: white adipose tissue (WAT) and brown adipose tissue (BAT). WAT cells are classically spherical and composed of single lipid droplets and few mitochondria, while BAT cells are composed of multiple small lipid droplets and a large number of mitochondria [6]. In rodents, WAT depots reside in the subcutaneous region underneath the skin and the visceral region around the vital organs, while the largest BAT depot is located in the interscapular region (iBAT) [7]. Some smaller BAT depots can also be found near the blood vessels in the cervical, axillary, and paravertebral regions as well as in proximity to the thoracic and abdominal viscera [8,9].

Functionally, WAT stores chemical energy in the form of fat and can secrete hormone peptides, such as adiponectin, to regulate whole-body metabolism. In contrast, BAT dissipates stored chemical energy in the form of heat through non-shivering thermogenesis, a process mediated by the uncoupling protein-1 (*Ucp1*) in the mitochondria [8,10,11,12]. With this unique function, BAT can maintain body temperature in a cold environment and potentially regulate energy expenditure. BAT can also secrete unique batokines and lipokines to regulate whole-body metabolism, for example, Fgf21 and 12, 13-diHOME [13,14]. Because early studies found that BAT depots were more abundant in rodents and infants, BAT was thought to have very limited thermogenic and metabolic roles in adult humans [15,16]. This view has recently been challenged by several independent reports of the existence of BAT in adult humans, thereby renewing interest in utilizing BAT to regulate energy expenditure and counteract obesity in adult humans [17,18,19]. Surprisingly, unlike mouse BAT depots, which are mainly found in the interscapular and subscapular regions, the majority of human BAT depots are found in the supraclavicular region [20,21,22]. These depots were therefore named supraclavicular BAT (scBAT) depots.

We and others identified several novel BAT depots in mouse embryos and adult mice that were not reported in earlier studies [23,24,25]. These depots, which were found in the supraclavicular region of the mouse neck, bear anatomical resemblance to the supraclavicular BAT observed in adult humans. We therefore refer to these BAT depots as mouse scBAT. Our previous studies further showed that the mouse scBAT depots possess high thermogenic potential and share a molecular signature with human scBAT [23]. Our studies provide a mouse model for studying scBAT to help us better understand the molecular regulation and the developmental lineage of human scBAT.

Environmental factors, such as temperature and diets, can greatly impact adipose tissue function and whole-body metabolism [26,27,28,29]. This is specifically true for BAT because BAT-mediated non-shivering thermogenesis is not active at thermoneutral temperature but can be rapidly activated by cold stimulation [30,31,32,33]. High-fat diet (HFD) is another factor that can impact the prevalence and the function of BAT in mice and humans [34,35,36]. As a first step toward understanding how environmental temperature and diet can impact mouse scBAT function, we compared scBAT activity to other adipose depots, including iBAT, inguinal WAT (iWAT), and epididymal WAT (eWAT), under cold exposure, thermoneutrality, and HFD feeding by evaluating gene expression and morphological characteristics. In addition to scBAT, humans also possess highly thermogenic BAT in the deep neck on the lateral side of the trachea and esophagus and surrounding major blood vessels, named the deep neck BAT (dnBAT) [37]. We have searched the mouse neck and identified BAT depots embedded in the similar anatomical location within the deep neck of the mice. We named this depot the mouse dnBAT.

## 2. Materials and Methods

### 2.1. Animal

All mice experiments conducted in the paper were approved by the Baylor College of Medicine (BCM) Institutional Animal Care and Use Committee (IACUC). All experiments were conducted in accordance with BCM guidelines and regulations. C57BL/6 mice were purchased from the BCM Center for Comparative Medicine production colonies. Mice were housed in the mouse vivarium at ambient temperature (22 °C) on a 12-h light/dark cycle and were fed ad libitum with regular diet (PicoLab Select Rodent 50 IF/6F, 5V5R, 14.9% energy from fat, LabDiet). Some mice were fed an HFD in which 42% of energy is from fat (TD.88137, western diet, high in saturated fatty acids and sucrose, ENVIGO) starting at age 5 weeks for 10 or 16 weeks. Some mice were housed at thermoneutral temperature right after weaning for 8 weeks and were fed either regular diet (RD) or 7 weeks of HFD. All experiments were conducted using male mice.

### 2.2. Adipose Tissue Dissection

iBAT, iWAT, and eWAT were isolated using previously published procedures [9]. scBAT was isolated according to our previously published method [23]. After removing the scBAT, the sternocleidomastoid muscle was removed to expose the most inner part of the neck, where the carotid artery and internal jugular vein lie lateral to the thyroid. Then, dnBAT was carefully peeled off from the surrounding artery and vein with a pair of fine forceps. After removal of all non-adipose tissues from iBAT, scBAT, dnBAT, iWAT, and eWAT, and WAT surrounding BAT depots, these adipose depots were processed for histological analyses or frozen in liquid nitrogen and stored in a −80 °C freezer for total RNA or protein extractions. BAT and WAT depot weights were recorded using a laboratory analytical scale. Adipose tissues were imaged by placing the tissue on an absorber pad on a Nikon SMZ1500 dissecting microscope and were photographed using the DS-Fi1 camera connected to the dissecting microscope.

### 2.3. Core Body Temperature Measurement

The body temperature was measured using an animal rectal probe connected to a TH-8 thermometer (Physitemp, Clifton, NJ, USA). Mice were housed at 4 °C for 6 h, and body temperature was measured every hour.

### 2.4. Histology

For histology, mouse adipose tissue samples were fixed in 4% paraformaldehyde for 48 h at 4 °C, washed with PBS, embedded in Paraffin, and sectioned. Hematoxylin and Eosin (H&E) staining was conducted using previously described procedures, and images were acquired using a Nikon microscope [23,38].

### 2.5. Real-Time Quantitative PCR (RT-qPCR)

Frozen adipose tissue was powdered in liquid nitrogen, mixed with 0.5–0.6 mL of PureZOL reagent (Bio-Rad, Hercules, CA, USA), and incubated for 5 min at room temperature. During the incubation, the powdered tissue was homogenized with a handheld pellet pestle grinder (Kimble) and syringe, and vortexed frequently. This was followed by adding 0.25 mL of chloroform (Thermo Fisher Scientific, Waltham, MA, USA) to the homogenized tissue and incubated for another 5 min at room temperature and vortexed frequently. The homogenized tissue was then centrifuged at 4 °C at 14,000 rpm for 20 min. To isolate total RNA, 200 µL of supernatant was transferred to a 1.5 mL microcentrifuge tube, and equal amounts of 70% ethanol were added into the same tube and mixed thoroughly. Then, 400 µL lysate was transferred to the spin column, which was centrifuged at 14,000 rpm for 2 min, and the flow through was discarded. The tissue lysate was further purified using an Aurum Total RNA Fatty and Fibrous Tissue Kit (Bio-Rad) according to the manufacturer’s instructions. After that, 500 ng^−1^ µg of total RNA was reverse transcribed into cDNA using dNTPs, Oligo(dT), and the SuperScript III Reverse Transcriptase (Thermo Fisher Scientific). RT-qPCR reactions were performed using the Powerup SYBR Green master mix (Thermo Fisher Scientific) in a CFX96 Touch Real-Time PCR Detection System (Bio-Rad). Relative mRNA expression level of each gene was normalized to the housekeeping gene β-actin. Primer sequences are listed in the Table S1.

### 2.6. Western Blot

Adipose tissues were frozen in liquid nitrogen, powdered with a pestle and mortar, and sonicated with a handheld pellet pestle grinder in standard RIPA buffer with protease inhibitors (1:200 dilution, Aprotinin, Pepstatin, Phenylmethylsulfonyl fluoride and protease inhibitor cocktail, Sigma-Aldrich, St. Louis, MO, USA) and phosphatase inhibitors (PhosSTOP, Roche Molecular Systems, Indianapolis, IN, USA). After sonication, protein lysates were incubated on ice for 10 min and centrifuged at 14,000 rpm at 4 °C for 15 min, and the supernatants were collected and stored in a −80 °C freezer. The protein concentration was determined with a Pierce BCA Protein Assay kit (Thermo Fisher Scientific). We separated 5–30 µg of protein lysate by SDS–PAGE and transferred onto a PVDF membrane using the eBlot L1 transfer system (GenScript, Piscataway, NJ, USA). The membrane was blocked with 5% milk/TBST buffer (or 3% BSA/TBST for p-HSL) overnight at 4 °C or 1 h at room temperature and incubated with primary antibodies overnight at 4 °C or 2 h at room temperature. The membrane was then incubated with the secondary antibody, anti-rabbit HRP (Jackson ImmunoResearch Laboratories, West Grove, PA, USA) at 1:10,000 dilution (or 1:3000 for β-actin) in 2.5% milk/TBST buffer (or TBST only for p-HSL), and developed using a Pierce ECL Plus Substrate Kit (Thermo Fisher Scientific). The primary antibodies used in this study included: rabbit anti-β-actin (1:2000 dilution in 5% milk/TBST, Cell Signaling 4967S), rabbit anti-p-HSL (1:1000 dilution in 3%BSA/TBST, Cell Signaling P-Ser^563^, 4139; P-Ser^565^, 4137T), rabbit anti-HSL (1:1000 dilution in 3%BSA/TBST, Cell Signaling 4107T), and rabbit anti-UCP1 (1:15,000 dilution in 3%BSA/TBST, Abcam ab10983). β-actin was used as a loading control.

### 2.7. Lipid Droplet Size Measurement

H&E-stained adipose tissue sections from RD- and HFD-treated mice and mice housed at ambient and thermoneutral temperatures were imaged at 400× magnification with a DS-Fi1 camera connected to a Nikon Eclipse 80i stereomicroscope. Three slides were randomly selected from each adipose tissue sample (scBAT, iBAT, iWAT, and eWAT) for lipid droplet size measurement using the area measurement tool from the ImageJ Software (version 1.53i). Each image was scaled to 0.09 μm/pixel, and the lipid droplets were marked manually using freehand selection from the ImageJ toolbar and quantified using the measure tool in the Analyze menu. All lipid droplets on the imaged slides were counted except the lipid droplets on the edges of the image that were partially cut off. To compare lipid droplet size, we grouped lipid droplets with similar sizes into three groups, small, medium, and large sizes, and calculated the percentage of small, medium, and large lipid droplets in RD-, HFD-treated mice and in mice housed at ambient and thermoneutral temperatures. More than 15,000 lipid droplets from scBAT and iBAT, more than 1000 lipid droplets from iWAT, and more than 400 lipid droplets from eWAT were measured from 3 independently collected samples.

### 2.8. Statistical Analysis

Statistical analysis was performed using unpaired two-tailed Student’s *t*-test for two group comparisons and One-way ANOVA for multiple group comparisons. We considered *p* values < 0.05 statistically significant. Statistical analyses were performed using GraphPad Prism 8 software. All data are presented as mean ± SEM.

## 3. Results

### 3.1. Histological and Molecular Analyses of Acute Cold Exposure Induced Changes in scBAT and Other Adipose Depots

**Figure 1:**
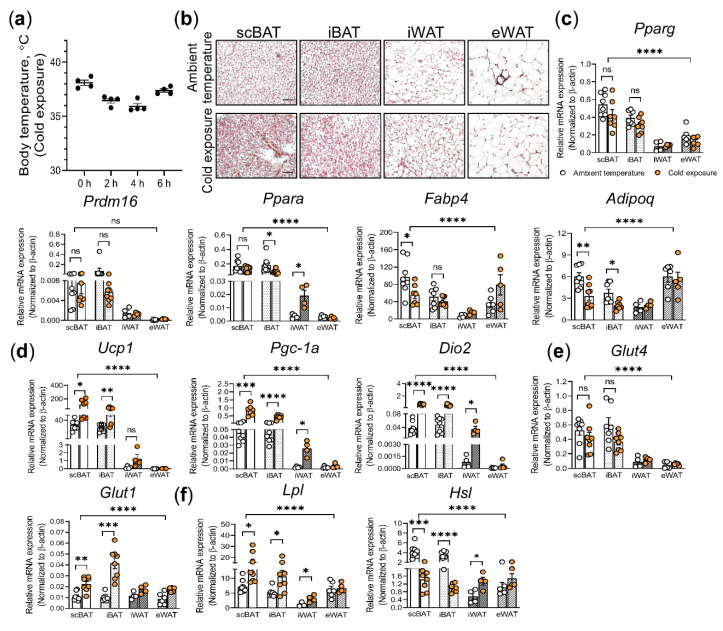
The effect of cold exposure on the adipocyte morphology and gene expression in scBAT, iBAT, iWAT, and eWAT. (**a**) The body temperatures of male C57BL/6 mice undergoing cold exposure (4 °C). *n* = 4. h: hour. (**b**) Representative images of the H&E stained sections of scBAT, iBAT, iWAT, and eWAT isolated from mice housed for 6 h at ambient temperature (22 °C) or exposed to cold (4 °C). *n* = 3. Scale bar = 50 µm. (**c–f**) RT-qPCR analysis of adipocyte differentiation marker genes, thermogenic genes, glucose uptake genes and lipid processing genes in scBAT, iBAT, iWAT, and eWAT depots from mice housed at ambient temperature and under acute cold exposure (4 °C for 6 h). (**c**) Adipocyte differentiation markers, *Pparg*, *Prdm16*, *Fabp4*, and *Adipoq*. (**d**) Thermogenic genes, *Ucp1*, *Pgc-1α*, and *Dio2*. (**e**) Glucose transporters, *Glut4* and *Glut1*. (**f**) Lipid uptake and processing genes, *Lpl* and *Hsl*. The expression of these genes is normalized to the housekeeping gene β-actin. Data are presented as mean ± SEM. *n* = 4–8 for each group. * *p* < 0.05, ** *p* < 0.01, *** *p* < 0.001, **** *p* < 0.0001, ns = nonsignificant.

To test the effect of cold exposure on scBAT and other adipose depots, the 8-week-old male C57BL/6 mice that had been housed at the ambient temperature (22 °C) and fed RD were subjected to cold exposure (4 °C) for 6 h. During cold exposure, mice were housed singly with food, water, and nestlet. The body temperature of these mice was measured every hour. The body temperature of these mice dropped 1–2 degrees below their normal body temperature shortly after cold exposure and recovered by the end of the experimental period (Figure 1a). These changes in body temperature suggested that these mice initially suffered from mild hypothermia, but thermogenesis was induced thereafter to maintain core body temperature. To determine whether scBAT was involved in this process, we isolated scBAT and other adipose depots, including iBAT, iWAT, and eWAT, from mice housed at ambient temperature and mice treated with cold exposure, and performed H&E staining and gene expression analyses. We first examined the overall morphology of adipocytes in H&E-stained adipose depot sections. Consistent with previously observed data, we found that H&E-stained scBAT and iBAT from mice housed at ambient temperature possessed brown adipocytes with multiple small lipid droplets, while iWAT and eWAT possessed white adipocytes with a single large lipid droplet (Figure 1b). In contrast, H&E stained-scBAT and iBAT from cold-exposed mice showed higher levels of red blood cell staining, much smaller brown adipocytes, and fewer lipid droplets, indicating that cold exposure induced a high volume of blood flow into these two adipose tissues and the loss of lipid droplets in the brown adipocytes (Figure 1b). Importantly, compared to iBAT, this cold-induced morphological change was more severe in scBAT as shown by even more intense red blood cell staining, smaller adipocyte size, and nearly complete loss of lipids (Figure 1b). A similar reduction in the adipocyte size was also seen in the H&E-stained iWAT and to a lesser extent in the eWAT (Figure 1b). Since glucose and lipids are the main fuels for BAT-mediated thermogenesis, the loss of lipid droplets in brown adipocytes and the increase of blood flow from cold-exposed mice indicates that scBAT and iBAT were actively utilizing the stored nutrients and recruiting more from the bloodstream to fuel thermogenesis [39]. To further confirm this, we next performed gene expression analysis to determine the expression of a set of genes involved in thermogenesis, including genes involved in brown adipocyte differentiation, thermogenesis, and nutrient utilization.

We first conducted RT-qPCR to examine the expression of genes involved in brown adipocyte differentiation, including Peroxisome Proliferator-Activated Receptor gamma (*Pparg*), *Pparα*, PR domain containing 16 (*Prdm16*), fatty acid binding protein 4 (*Fabp4*), and adiponectin (*Adipoq*) [40,41,42,43,44,45]. Consistent with previously reported data [44,46,47,48] showing expression of *Pparg*, *Prdm16*, and *Pparα* is higher in BAT than in WAT, these genes were also expressed higher in BAT than in WAT from the mice housed in ambient and cold temperatures (Figure 1c). When comparing scBAT to iBAT, we found that *Pparg*, *Prdm16* and *Pparα* were expressed at similar levels in mice housed at ambient and cold temperatures, while *Fabp4*, and *Adipoq* were expressed at slightly lower levels in scBAT from mice housed in cold temperature (Figure 1c). Together, these data suggest that the effect of acute cold exposure on brown adipocyte differentiation is minimal. Additionally, we observed an increase in the expression of *Pparα* in iWAT from cold-exposed mice, which is consistent with previous studies [49,50]. We next examined the expression of genes involved in BAT-mediated thermogenesis, including *Ucp1*, peroxisome proliferator-activated receptor-gamma coactivator- 1alpha (*Pgc-1α*), and the type 2 iodothyronine deiodinase (*Dio2*) [51,52,53,54]. Our analysis showed that the expression of *Ucp1*, *Pgc-1α*, and *Dio2* were significantly increased in BAT depots with higher expression of these three genes in scBAT than in iBAT in cold-exposed mice (Figure 1d). However, while the expression level of these three genes increased, UCP1 protein levels did not change after 6 h of cold exposure (Figure S1a). Additionally, we observed an increase of these three genes in iWAT but not in eWAT in cold-exposed mice.

To further examine the effect of cold exposure on scBAT, we next analyzed the expression of genes known to mediate glucose uptake in adipose tissues, including glucose transporter type 1 (*Glut1*) and 4 (*Glut4*) [55,56]. In agreement with previous analyses, we found that *Glut4* was more abundantly expressed in BAT than in WAT [57]. However, the cold exposure did not induce a notable change in *Glut4* expression in any of the adipose depots examined as previously reported (Figure 1e). Interestingly, the expression of *Glut1* was consistently higher in iBAT than in scBAT in cold-exposed mice compared to mice housed at ambient temperature (Figure 1e).

Lastly, we examined lipid processing in scBAT by analyzing the expression of lipoprotein lipase (*Lpl*), a protein that recruits the circulating fatty acid to BAT, and hormone-sensitive lipase (*Hsl*), a rate-limiting enzyme of lipolysis [58,59]. Cold exposure induced an increase in *Lpl* expression in both scBAT and iBAT with the induction in its expression being higher in scBAT than in iBAT (Figure 1f). Contrary to *Lpl* expression, *Hsl* expression was slightly reduced after cold exposure in scBAT and further reduced in iBAT. Meanwhile, *Hsl* expression was slightly increased in WAT (Figure 1f). This reduction in *HSL* in BAT was also seen at the protein level; specifically, phospho-HSL (p-HSL) [60] was similarly slightly reduced in scBAT and further reduced in iBAT after cold exposure (Figure S1b).

### 3.2. The Effect of HFD on the Adipocyte Morphology and Gene Expression in scBAT and Other Adipose Depots

**Figure 2:**
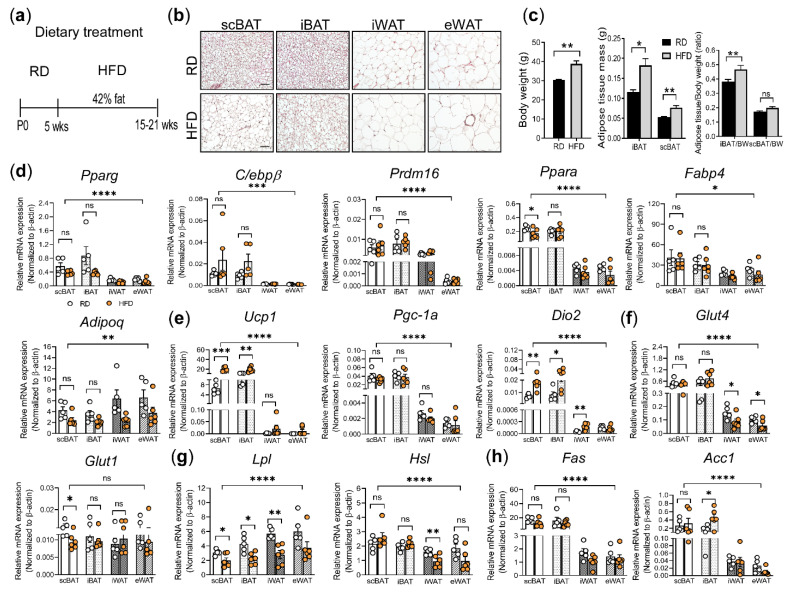
The effect of HFD on the adipocyte morphology and gene expression in scBAT, iBAT, iWAT, and eWAT. (**a**) Schematic diagram of the HFD study design. P0: postnatal day 0, wks: weeks. Mice were subjected to 10 to 16 weeks of RD or HFD. (**b**) Representative images of H&E-stained sections of scBAT, iBAT, iWAT, and eWAT from mice treated with RD or HFD for 16 weeks. *n* = 3–4. Scale bar = 50 µm. (**c**) Body weight (BW), adipose tissue mass, and ratio of adipose tissue to body weight of mice described in (**b**) (RD, *n* = 4 and HFD, *n* = 4). (**d**–**h**) RT-qPCR analysis of adipocyte differentiation marker genes, thermogenic genes, glucose uptake genes, and lipid processing genes in scBAT, iBAT, iWAT, and eWAT depots from mice fed 10 weeks with RD or 10 weeks with HFD. (**d**) Adipocyte differentiation markers, *Pparg*, *C/ebpβ*, *Prdm16*, *Pparα*, *Fabp4*, and *Adipoq*. (**e**) Thermogenic genes, *Ucp1*, *Pgc-1α*, and *Dio2*. (**f**) Glucose transporters, *Glut4* and *Glut1*. (**g**) Lipid uptake and processing genes, *Lpl* and *Hsl*. (**h**) Lipogenesis genes, *Fas* and *Acc1*. Data are presented as mean ± SEM. The expression of these genes is normalized to the housekeeping gene β-actin. *n* = 5–6 for each group. * *p* < 0.05, ** *p* < 0.01, *** *p* < 0.001, **** *p* < 0.0001, ns = nonsignificant.

To test the effect of high-fat diet (HFD) on scBAT activity, 5-week-old male C57BL/6 mice that were housed at ambient temperature were fed with RD or HFD (42% of energy from fat) for 10 weeks or 16 weeks (Figure 2a). The adipose tissues were collected from these mice and processed for H&E staining and gene expression analysis. Compared to adipose tissue in H&E-stained sections from RD-fed mice, adipose tissue from 16-week HFD-fed mice showed an increase in adipocyte size with the appearance of more large lipid droplets in both BAT and WAT (Figure 2b and Figure S2). In addition, multilocular brown adipocytes had undergone morphological changes with the appearance of single lipid droplets, resembling WAT (Figure 2b). Importantly, the degree of HFD-induced morphological change was more prominent in scBAT than in iBAT. To further determine the effect of HFD on BAT depots, we compared the adipose tissue mass between scBAT and iBAT after 10 weeks of HFD treatment. Our analysis showed that HFD feeding induced an overall body weight gain and increase in BAT mass compared to RD-fed mice. Interestingly, BAT mass increased more in iBAT than in scBAT, and the ratio of adipose tissue/body weight was much higher in iBAT than in scBAT (Figure 2c). These results collectively suggest that HFD affects scBAT and iBAT differently, with HFD inducing more prominent changes in adipocyte morphology in scBAT while increasing depot mass in iBAT.

In addition to morphological analysis, we also performed gene expression analysis to determine the expression of a panel of genes involved in brown adipocyte differentiation, including *Pparg*, CCAAT/enhancer binding protein beta (*C/ebpβ*), *Prdm16*, *Pparα*, *Fabp4*, and *Adipoq*, in scBAT, iBAT, iWAT, and eWAT isolated from mice after 10 weeks of RD and HFD feeding. Our analysis showed that the expression of these genes was relatively similar in both BAT and WAT from RD and HFD-fed mice with one exception, *Pparα*, which was expressed at a slightly lower level in scBAT in HFD-fed mice (Figure 2d). Interestingly, while there was no significant difference in the expression of these brown adipocyte-differentiation-specific genes, the expression of genes involved in thermogenesis, including *Ucp1* and *Dio2*, significantly increased in scBAT and to a lesser extent in iBAT from HFD-fed mice compared to RD-fed mice (Figure 2e). The increase in *Ucp1* expression in iBAT in HFD-fed mice has been reported by many groups [61]. Our analysis showed that, as with iBAT, *Ucp1* expression also increased in scBAT after HFD-feeding in mice. However, the expression of *Pgc-1α* in scBAT and iBAT between the HFD and RD mice remained similar under our current HFD regime.

While *Glut4* was expressed at similar levels in scBAT and iBAT from RD or HFD-fed mice, its expression was notably reduced in WAT in HFD-fed mice compared to RD-fed mice (Figure 2f). Unlike *Glut4*, *Glut1* expression was slightly reduced in scBAT but not in iBAT or WAT depots (Figure 2f). Since adipocytes are the main cell type that process and store lipids, we next analyzed the expression of genes involved in lipid uptake, *Lpl*, and lipolysis, *Hsl*. The expression of *Lpl* was reduced in scBAT, iBAT, and iWAT depots from HFD-fed mice, suggesting that 10 weeks of HFD-feeding may suppress its expression in these depots. The expression of *Hsl* was only slightly reduced in iWAT, and no detectable changes in scBAT, iBAT and eWAT were observed between HFD and RD-fed mice (Figure 2g). Finally, we examined the expression of two essential genes in de novo lipogenesis, fatty acid synthase (*Fas*) and Acetyl-CoA carboxylase 1 (*Acc1*), which convert dietary carbohydrate into fat [62,63]. In agreement with earlier studies [57,64], both *Fas* and *Acc1* were highly expressed in scBAT and iBAT compared to WAT. However, while it was reported that HFD can suppress de novo lipogenesis in BAT [65], our analysis showed a slight increase of *Acc1* in iBAT and no significant changes in the expression of *Fas* and *Acc1* in other adipose tissues under our HFD regime (Figure 2h). We reasoned that this discrepancy may result from the difference between the percentage of fat content and duration of HFD feeding used in our study and others.

In summary, while there were minor changes in the expression of genes involved in nutrient uptake and processing, the genes that were affected the most by the HFD feeding are those involved in BAT-mediated thermogenesis. Specifically, both *Ucp1* and *Dio2* expression were increased in scBAT and iBAT from HFD-fed mice compared to RD-fed mice, suggesting that HFD induces the expression of thermogenesis-associated genes in BAT [66,67,68].

### 3.3. Histological and Gene Expression Analysis of Thermoneutrality Induced Changes in scBAT and Other Adipose Depots

**Figure 3:**
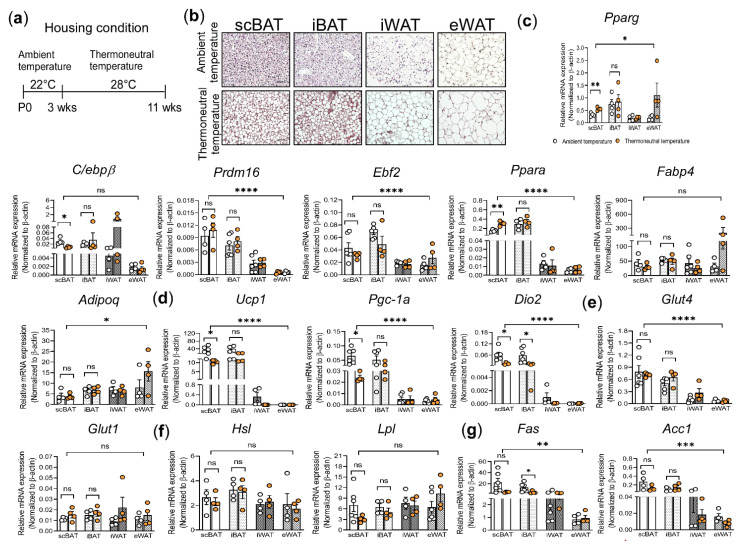
The effect of thermoneutrality on the adipocyte morphology and gene expression in scBAT, iBAT, iWAT, and eWAT. (**a**) Schematic diagram of the thermoneutral housing study design. Mice were housed at the thermoneutral temperature for 8 weeks. (**b**) Representative images of H&E-stained sections of scBAT, iBAT, iWAT, and eWAT from mice housed under ambient and thermoneutral temperatures. *n* = 3. Scale bar = 50 µm. (**c**–**g**) RT-qPCR analysis of adipocyte differentiation marker genes, thermogenic genes, glucose uptake genes, and lipid-processing genes in scBAT, iBAT, iWAT, and eWAT depots from mice housed under ambient and thermoneutral temperatures. (**c**) Adipocyte differentiation markers, *Pparg*, *C/ebpβ*, *Ebf2*, *Prdm16*, *Pparα*, *Fabp4*, and *Adipoq*. (**d**) Thermogenic genes, *Ucp1*, *Pgc-1α*, and *Dio2*. (**e**) Glucose transporters, *Gult4* and *Glut1*. (**f**) Lipid uptake and processing genes, *Lpl* and *Hsl*. (**g**) Lipogenesis genes, *Fas* and *Acc1*. The expression of these genes is normalized to the housekeeping gene β-actin. Data are presented as mean ± SEM. *n* = 4–6. for each group. * *p* < 0.05, ** *p* < 0.01, *** *p* < 0.001, **** *p* < 0.0001, ns = nonsignificant.

The ambient temperature for housing laboratory mice in the vivarium is ~22 °C, which is below their thermoneutral temperature (28–30 °C) [26]. At this ambient temperature, mice are mildly cold-stressed, and, consequently, BAT-mediated thermogenesis is activated [27,69]. To probe whether housing mice at thermoneutrality affects scBAT activity, we housed 3-week-old male mice at thermoneutral temperature or at ambient temperature fed ad libitum RD for 8 weeks (Figure 3a). We then processed the 4 adipose depots from these mice for H&E staining and gene expression analyses. Compared to mice housed at ambient temperature, lipid droplets in both brown and white adipocytes were much larger in mice housed at thermoneutral temperature (Figure 3b and Figure S3). Additionally, we found that some brown adipocytes in mice housed at thermoneutral temperature possess a unilocular lipid droplet that resembles those of the white adipocytes, a sign of whitening of the brown adipocytes (Figure 3b). This observation is in line with previous findings that brown adipocytes showed signs of whitening when housed at thermoneutrality [70]. Interestingly, our analysis showed that more brown adipocytes appeared unilocular in scBAT than iBAT, suggesting that scBAT undergoes a more advanced brown adipocyte whitening than iBAT.

To further determine the cause underlying this morphological change, we compared the expression of genes, including, *Pparg*, *C/ebpβ*, early B cell factor-2 (*Ebf2*), *Prdm16*, *Pparα*, *Fabp4*, and *Adipoq*, involved in brown adipocyte differentiation in adipose depots isolated from mice housed at ambient temperature to the mice housed at thermoneutral temperature. The expression of *Pparg* and *Pparα* was slightly increased in scBAT compared to other depots from mice housed at thermoneutral temperature relative to those housed at ambient temperature. However, the expression of *C/ebpβ* was slightly reduced in scBAT but increased in iWAT from mice housed at thermoneutral temperature relative to those housed at ambient temperature (Figure 3c). Additionally, the expression of *Prdm16*, *Ebf2*, *Fabp4*, and *Adipoq* were not significantly different in BAT and WAT from mice housed at thermoneutral compared to ambient temperature (Figure 3c). Together, these analyses suggest that housing mice at thermoneutral temperature for 8 weeks has no significant effect on the expression of genes involved in adipocyte differentiation.

We next compared the expression of genes involved in BAT-mediated thermogenesis in adipose depots isolated from mice housed at ambient temperature to the mice housed at thermoneutral temperature. Compared to mice housed at ambient temperature, mice housed at thermoneutral temperature showed significantly decreased *Ucp1* and *Pgc-1α* expression in scBAT but not in iBAT, indicating that thermogenesis was greatly suppressed in scBAT at thermoneutral temperature (Figure 3d). While the expression of *Ucp1* and *Pgc-1α* was not significantly changed, the expression of *Dio2* was decreased in the iBAT, suggesting that, to a lesser extent, thermogenesis was also suppressed in iBAT (Figure 3d). These data were consistent with our morphological analyses, indicating a more severe whitening of scBAT compared to iBAT under thermoneutrality.

Lastly, we compared the expression of genes involved in nutrient uptake and processing in adipocytes. The expression of these genes, including *Glut4*, *Glut1*, *Lpl*, and *Hsl*, was not significantly different between mice housed at ambient and thermoneutral temperatures (Figure 3e,f). Although it was not statistically significant, we also found that the expression of *Fas* and *Acc1* was reduced in scBAT at thermoneutrality and that this reduction was less notable in iBAT (Figure 3g). This observation is consistent with previous studies which indicate that de novo lipogenesis was suppressed in iBAT from mice housed at thermoneutrality [71]. Together, these results showed that housing mice under thermoneutral conditions for 8 weeks has a minimal effect on the expression of genes involved in adipocyte differentiation and nutrient processing. However, under this housing condition, the expression of genes involved in thermogenesis was significantly reduced, and the degree of reduction is greater in scBAT than in iBAT.

### 3.4. Histological and Molecular Analyses of HFD Induced Changes in scBAT and Other Adipose Depots under Thermoneutrality

**Figure 4:**
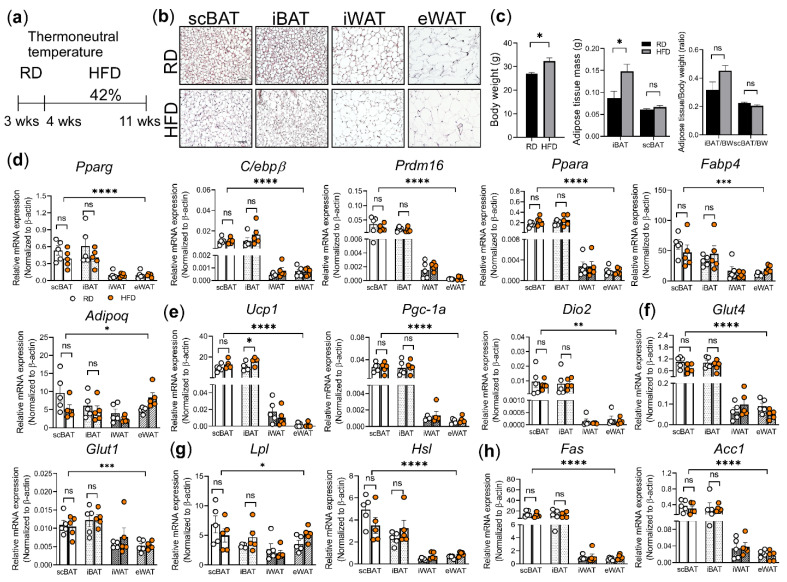
The effect of HFD on the adipocyte morphology and gene expression in scBAT, iBAT, iWAT, and eWAT under thermoneutrality. (**a**) Schematic diagram of the thermoneutral housing study design under HFD. 3-week-old mice were housed at 28 °C for 8 weeks. Mice were fed with RD for 1week for adaptation and then fed with HFD for an additional 7 weeks. (**b**) Representative images of H&E-stained sections of scBAT, iBAT, iWAT, and eWAT from mice fed RD or HFD under thermoneutral temperatures. *n* = 5. Scale bar = 50 µm. (**c**) Body weight, adipose tissue mass, and ratio of adipose tissue to body weight of mice described in B (RD, *n* = 5 and HFD, *n* = 5). (**d**–**h**) RT-qPCR analysis of adipocyte differentiation marker genes, thermogenic genes, glucose uptake genes, and lipid processing genes, and lipogenesis genes in scBAT, iBAT, iWAT, and eWAT depots from mice housed under ambient and thermoneutral temperatures. (**d**) Adipocyte differentiation markers, *Pparg*, *C/ebpβ*, *Prdm16*, *Pparα*, *Fabp4*, and *Adipoq*. (**e**) Thermogenic genes, *Ucp1*, *Pgc-1α*, and *Dio2*. (**f**) Glucose transporters, *Glut4* and *Glut1*. (**g**) Lipid uptake and processing genes, *Lpl* and *Hsl*. (**h**) Lipogenesis genes, *Fas*, *Acc1*. The expression of these genes is normalized to the housekeeping gene β-actin. Data are presented as mean ± SEM. *n* = 5 for each group. * *p* < 0.05, ** *p* < 0.01, *** *p* < 0.001, **** *p* < 0.0001, ns = nonsignificant.

Both we and others found that mice housed at thermoneutrality can reduce BAT-mediated thermogenesis [72]. We next tested whether this reduction is further enhanced by HFD feeding under thermoneutrality. To test this, we first housed 3-week- old male mice at thermoneutral temperature and fed them with RD for one week while they adapted to the housing temperature change. After 1 week, we randomly divided these mice into 2 groups: 1 group fed RD and the other group fed HFD for an additional 7 weeks (Figure 4a). At the end of 8 weeks, we isolated scBAT, iBAT, iWAT, and eWAT from these mice and performed H&E staining and gene expression analyses.

As shown in Figure 3b, under the thermoneutral housing condition, BAT undergoes a morphological change in which some adipocytes become large and lose their multilocular lipid droplet character. Brown adipocytes in HFD-fed mice housed at thermoneutrality were further enlarged and had more unilocular lipid droplets compared to RD-fed mice (Figure 4b). Similarly, white adipocyte size was also further enlarged in mice fed HFD than RD at thermoneutrality (Figure 4b). In addition to morphology, adipose tissue mass between scBAT and iBAT was also compared to distinguish the effects of HFD on scBAT and iBAT. We first confirmed that 7 weeks of HFD results in greater body weight-gain compared to RD-fed mice (Figure 4c). The mass and ratio of adipose tissue/body weight were also higher in iBAT than in scBAT (Figure 4c). Even though the HFD regime that we implemented at thermoneutrality (7 weeks of HFD) was shorter than the one implemented at ambient temperature (10 weeks of HFD) (Figure 2c), we observed a similar trend in which HFD did not induce as large an increase in scBAT depot mass as it did in iBAT, thereby further supporting the notion that HFD has different effects on scBAT and iBAT.

Besides morphological analysis, we also compared the expression of genes involved in brown adipocyte differentiation, including *Pparg*, *C/ebpβ*, *Prdm16*, *Pparα*, *Fabp4*, and *Adipoq*. Our analysis showed that the expression of these genes in scBAT and iBAT was relatively similar between HFD and RD-fed mice at thermoneutrality, suggesting that 7 weeks of HFD feeding at thermoneutrality did not further alter the expression level of these genes (Figure 4d). Next, we examined the expression of genes involved in BAT-mediated thermogenesis. While we did not observe a further increase in *Ucp1* expression in scBAT, we did find that *Ucp1* expression in iBAT from mice fed HFD slightly increased compared to mice fed RD. This increase is in agreement with previous studies on iBAT [61,73,74]. However, unlike *Ucp1*, there is no significant difference in the expression of *Pgc-1α*, and *Dio2* in mice fed HFD compared to those fed RD (Figure 4e).

Next, we examined the expression of genes involved in nutrition uptake and processing. Both *Glut4* and *Glut1* were expressed at similar levels in mice fed HFD or RD at thermoneutrality, suggesting that glucose uptake mediated by these two genes was not affected by HFD at thermoneutrality in the short term (Figure 4f). A similar trend was observed for the expression of *Lpl*, *Hsl*, *Fas*, and *Acc1* under the same HFD regime (Figure 4g,h). Overall, our molecular analyses suggest that the thermogenic potential of scBAT was not significantly changed in mice fed HFD for 7 weeks at thermoneutrality as shown by the unchanged expression of *Ucp1*, *Pgc-1α*, and *Dio2* in these mice compared to RD-fed mice under thermoneutrality.

### 3.5. Histological and Molecular Analysis of dnBAT Exposed to Different Environmental Temperatures

**Figure 5:**
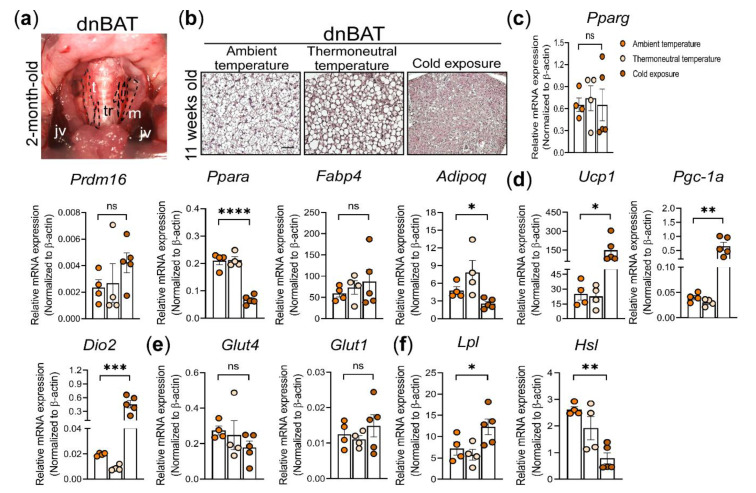
The effect of thermoneutrality and cold exposure on the adipocyte morphology and gene expression of dnBAT in C57BL/6 mice. (**a**) Representative image of the anatomical location of dnBAT in 2-month-old mice. tr: trachea, t: thyroid, m: sternocleidomastoid muscle, jv: external jugular vein. dnBAT is outlined by the black dotted line. (**b**) Representative images of H&E-stained sections of dnBAT from mice housed at ambient, thermoneutral, and cold temperatures. *n* = 3. Scale bar = 50 µm. (**c**–**f**). RT-qPCR analysis of adipocyte differentiation marker genes, thermogenic genes, glucose uptake genes, and lipid-processing genes in scBAT, iBAT, iWAT, and eWAT depots from mice housed at ambient and thermoneutral temperatures and under acute cold exposure. (**c**) Adipocyte differentiation markers, *Pparg*, *Prdm16*, *Fabp4*, and *Adipoq*. (**d**) Thermogenic genes, *Ucp1*, *Pgc-1α*, and *Dio2*. (**e**) Glucose transporters, *Glut4* and *Glut1*. (**f**) Lipid uptake and processing genes, *Lpl* and *Hsl*. The expression of these genes is normalized to the housekeeping gene β-actin. Data are presented as mean ± SEM. *n* = 4–5 for each group. * *p* < 0.05, ** *p* < 0.01, *** *p* < 0.001, **** *p* < 0.0001, ns = nonsignificant.

In addition to the BAT in the supraclavicular region, BAT depots located in the most inner parts of the neck in adult humans, collectively named the deep neck BAT, have been described [75,76]. As with the scBAT, these depots also possess high thermogenic potential. To determine whether mice possess BAT depots in the similar anatomical location, we removed the sternocleidomastoid muscle and found a very thin layer of brownish adipose tissue situated next to the thyroid, surrounding the carotid arteries. This thin layer of adipose tissue is located symmetrically on both sides of the neck and extends from the ventral neck toward the lateral neck. Since this brownish adipose depot is located in the similar anatomical location as human deep neck BAT, we named this depot the mouse deep neck brown adipose tissue (dnBAT) depot (Figure 5a). Compared to scBAT and iBAT, dnBAT is much smaller (Figure S4). To determine whether the mouse dnBAT is thermogenic, we isolated dnBAT from both sides of the neck from mice housed at ambient, thermoneutral, and cold temperatures for histological and gene expression analyses. H&E staining showed that the dnBAT depots, like scBAT and iBAT, histologically resemble the classic BAT and are composed of adipocytes with multiple small lipid droplets (Figure 5b). Lipid droplets in dnBAT were larger in mice housed at thermoneutrality and became much smaller in mice exposed to cold (Figure 5b). The reduction in the size of the lipid droplets is one of the indicators that brown adipocytes are dissipating lipids in the form of heat. Our histological analyses indicated that dnBAT is another previously uncharacterized BAT depot with high thermogenic potential in mice.

To further understand dnBAT’s thermogenic potential, we again examined a set of genes involved in brown adipocyte differentiation, thermogenesis, and nutrient processing in dnBAT from mice housed at ambient, thermoneutral, and cold temperatures. Our analysis indicated that the genes involved in brown adipocyte differentiation, including *Pparg*, *Prdm16*, *Pparα*, *Adipoq*, and *Fabp4*, were expressed at similar levels in dnBAT between mice housed at ambient and thermoneutral temperatures. Interestingly, we also found that the expression of *Prdm16* was slightly increased and the expression of *Pparα* was reduced in dnBAT from cold-exposed mice (Figure 5c). In contrast to their expression in dnBAT, the expression of these two genes was not significantly different in scBAT or iBAT from the cold-exposed mice. Collectively, this analysis suggests that brown adipocyte differentiation markers are differentially expressed in anatomically different BAT depots in cold-exposed mice. Next, we examined the expression of genes involved in BAT-mediated thermogenesis in dnBAT. The expression of *Ucp1*, *Pgc-1α*, and *Dio2* in dnBAT was similar for mice housed at both ambient and thermoneutral temperatures (Figure 5d). Consistent with the expression in scBAT and iBAT, *Ucp1*, *Pgc-1α*, and *Dio2* were significantly upregulated in dnBAT from acute cold-exposed mice, indicating that dnBAT also possesses a high thermogenic potential (Figure 5d).

Lastly, to probe how nutrients may be utilized in dnBAT, we examined the expression of *Glut1*, *Glut4*, *Lpl*, and *Hsl* in dnBAT from mice housed at ambient, thermoneutral, and cold temperatures. *Glut4* and *Glut1* expression in dnBAT was similar for these three different housing temperatures (Figure 5e). As in scBAT, the expression of *Lpl* was increased but *Hsl* was reduced in dnBAT from cold-exposed mice (Figure 5f). Together, our histological and gene expression analyses showed that dnBAT is a thermogenic BAT depot like its counterpart in adult humans. With its important role in the regulation of thermogenesis in humans, more studies on the mouse dnBAT are warranted.

## 4. Discussion

We recently identified mouse scBAT that is anatomically analogous to human scBAT [21]. Here, we report the first in a series of genetic and molecular studies designed to understand the function of scBAT by probing how environmental factors affect scBAT activity.

Studies using iBAT have revealed that BAT function can be greatly influenced by environmental factors [77,78,79,80]. Among these factors, the most influential one is the environmental temperature, with BAT-mediated thermogenesis being activated by cold temperature and inactivated at thermoneutrality. By conducting H&E staining and gene expression analysis, we found that, although both scBAT and iBAT can mediate non-shivering thermogenesis, differences existed between scBAT and iBAT. Most notably, we found that 6 h of cold exposure can induce morphological changes, including increased blood flow and depletion of adipocyte stored lipids, and induce higher expression of thermogenic genes in scBAT than in iBAT. Together, these data suggest that scBAT is at least as sensitive as iBAT or perhaps even more sensitive to cold exposure than iBAT. Interestingly, while we observed that the *Ucp1* transcript was more highly expressed in scBAT, the UCP1 protein was expressed at similar levels in scBAT and iBAT after 6 h of cold exposure. Several factors may contribute to this discrepancy between *Ucp1* transcript and protein levels. First, 6 h of cold exposure may be sufficient to induce an increase in *Ucp1* transcripts, but more time is needed to synthesize UCP1 protein. Another possibility is that the UCP1 protein abundance is subject to post-translational modification that is independent of its transcriptional regulation. Lastly, it could be that our western blotting is not sensitive enough to detect small changes at the protein level. It has been reported that the change in the expression of *Ucp1* transcript does not directly correlate to the change in its protein level during cold acclimation from thermoneutral temperature to cold [81]. While we observed similar UCP1 protein expression trends in scBAT and iBAT under our acute cold exposure, more molecular studies are needed in the future to determine if there are differences in the thermogenic potentials of *Ucp1*-mediated thermogenesis between scBAT and iBAT.

Additionally, while BAT depots rely on both fatty acids and glucose to fuel thermogenesis, we found that scBAT and iBAT may have different nutrient preferences under our cold exposure regimen. As indicated by our expression analyses, *Hsl* expression is reduced in scBAT and iBAT after 6 h of cold exposure. *Lpl* expression is induced more highly in scBAT, while *Glut1* expression is increased more in iBAT. These differences in changes in expression of genes involved in nutrient processing led us to speculate that mouse scBAT, and perhaps dnBAT as well, may rely more on circulating than intracellular fatty acids through the action of *Lpl*, while iBAT may rely more on *Glut1*-mediated glucose uptake to fuel thermogenesis under our cold exposure regimen. Whether these differences in *Lpl* and *Glut1* expression occur because of intrinsic regulation, such as distinct developmental origins, or are due to the anatomical locations or depot size differences between scBAT and iBAT needs to be addressed in future studies with genetic tracing studies, and more lipogenic and lipid uptake marker analyses and functional assays. Recent studies showed that fatty acids released from lipid droplets within BAT may not be essential substrates for BAT mediated thermogenesis during cold exposure because mice with deletion in genes involved in lipolysis, *ATGL* and *CGI-58*, and genes involved in triglyceride synthesis, *DGAT1* and *DGAT2*, in BAT are cold tolerant [82,83,84]. While these studies analyzed mainly iBAT, it would be of great interest to analyze scBAT in these mice.

To further understand how environmental temperature impacts scBAT activity, we also housed mice at thermoneutral temperature. As expected, both scBAT and iBAT undergo morphological changes to resemble WAT. Furthermore, the expression of thermogenic genes, including *Ucp1*, *Pgc-1α*, and *Dio2*, was reduced in scBAT and to a lesser extent in iBAT in mice housed at thermoneutral temperature compared to mice housed at the ambient temperature. Together, these data indicate a persistent decline of BAT thermogenic activity at thermoneutrality. Interestingly, in all the samples we examined, we consistently observed that the expression of thermogenic genes is reduced more in scBAT than in iBAT, indicating that scBAT is likely more sensitive to thermoneutrality-induced changes in thermogenic activity than iBAT. Currently, our knowledge on how thermoneutrality impacts BAT function is still very limited. As humans are born and live in a thermoneutral environment, it is critical to investigate the molecular mechanisms underlying these changes, and mouse scBAT is a suitable animal model for these purposes.

HFD feeding can induce adipose tissue dysregulation, including adipose tissue morphological remodeling in which both adipocyte size and depot mass are increased. In this regard, our analysis found there is a morphological difference between scBAT and iBAT in response to HFD feeding. Specifically, we found that under either ambient or thermoneutral temperature, HFD induced a more visible increase in adipocyte size (through increased of lipid droplet size) in scBAT without a significant increase in depot mass, whereas adipocyte size and mass were both increased in iBAT. In WAT, the adipose mass expansion can be induced through increases in lipid storage (hypertrophy) and in the number of new adipocytes (hyperplasia) [85]. Currently, it is not clear whether HFD-induced iBAT mass expansion is due to the addition of new brown adipocytes. Nevertheless, the differences in HFD-induced increase in mass expansion between scBAT and iBAT may indicate that the molecular mechanisms that regulate depot mass expansion are different for these two depots, which is worthy of further investigation considering that scBAT also exists in humans. Under our HFD regimes, the most notable change at the gene expression level is the increase of *Ucp1* expression in scBAT and iBAT. Expression showed a greater increase in scBAT than iBAT at ambient temperature. This observation is very intriguing and contradictory, as obesity can impair BAT-mediated thermogenesis in mice [86,87]. However, obesity induced by HFD feeding is also known to induce *Ucp1* expression in iBAT, and our finding confirms a similar increase in scBAT in mice housed at ambient temperature. While the underlying mechanism of this HFD-related increase in *Ucp1* expression has not been established, it has been speculated that fatty acids, specifically polyunsaturated fatty acids, may act directly or indirectly to activate *Ucp1* expression [88,89].

Interest in applying BAT-mediated thermogenesis to regulate energy expenditure and combat obesity was reignited after the rediscovery of thermogenic BAT in adult humans over a decade ago. While many sophisticated studies have been conducted for human scBAT, a tissue model for conducting genetic and molecular studies is needed to uncover more mechanistic insights regarding human scBAT function. Currently, iBAT is the most studied BAT in mice. However, this depot is anatomically different from the human scBAT. As we have shown in here, iBAT and scBAT responses to environmental stimuli are not identical, indicating there are some intrinsic differences between these two depots. These differences need to be taken into consideration when interpreting functional aspects of human scBAT using iBAT as a tissue model. Although we only applied simple histological and expression assays to probe the difference between scBAT and other adipose depots, the results obtained from this study can serve as a starting point for future comprehensive studies to understand the molecular function of scBAT in mice and humans under physiological conditions and during metabolic dysregulation.

While scBAT is the largest and the most studied BAT depot in humans, there are some smaller BAT depots that have not been extensively characterized in humans. In the most inner neck, a thin layer of BAT, the dnBAT, was first identified in humans, and here we describe an anatomically similar thermogenic dnBAT in mice. The neck, which is a small and narrow anatomical region, seems to be enriched in BAT. Interestingly, both in humans and mice, these neck BAT depots reside proximal to the major veins and arteries. In addition to further characterizing the molecular regulation of neck BAT, whether this unique anatomical feature of neck BAT depots has a significant physiological role in humans and mice needs to be studied.

## Data Availability

Not applicable.

## Supplementary Materials

The following are available online at https://www.mdpi.com/article/10.3390/cells10061370/s1, Table S1: Primer sequences for RT-qPCR, Figure S1: Protein levels of UCP1, HSL, and p-HSL in adipose tissues from mice housed at ambient temperature or after 6 h of cold exposure, Figure S2: Size distributions for lipid droplets in scBAT, iBAT, iWAT, and eWAT from mice fed RD or 16 weeks of HFD, Figure S3: Size distributions for lipid droplets in scBAT, iBAT, iWAT, and eWAT from mice housed at ambient or thermoneutral temperature for 8 weeks, Figure S4: Adipose tissue images.
