# Supplementary material for: Gene Expression Analysis of Environmental Temperature and High-Fat Diet-Induced Changes in Mouse Supraclavicular Brown Adipose Tissue
Source: Cells. 2021 Jun 2;10(6):1370. doi: 10.3390/cells10061370 (PMC8226907; doi:10.3390/cells10061370)
Supplement: Supplementary file 1 [file cells-10-01370-s001.zip › cells-1140020-supplementary.pdf]

Table S1. Primer sequences for RT-qPCR

| Gene | Forward primer (5'-3') | Reverse primer (5'-3') |
|---|---|---|
| *β-actin* | GGCACCACACCTTCTACAATGAGCT | GACAGCACAGCCTGGATGGCTAC |
| *Acc1* | GCAGTGTGGGCTGGCTGGG | ACATGGCCTGGCTTGGAGGG |
| *Adipoq* | CGTGATGGCAGAGATGGCACTC | AGCGATACACATAAGCGGCTTCTCC |
| *C/ebpβ* | GGACAAGCTGAGCGACGAGTACAAG | TGCTTGAACAAGTTCCGCAGGG |
| *Dio2* | TGTGTCTGGAACAGCTTCCTCCTAGA | AAGTCAAGAAGGTGGCATTCGGC |
| *Ebf2* | GCATTTCAGAGTCCACACAAGGAAATAA | ATGCTGTTGCTGGAGGTGCTGTAAT |
| *Fabp4* | ACACCGAGATTTCCTTCAAACTG | CCATCTAGGGTTATGATGCTCTTCA |
| *Fas* | CGCTGCGGAAACTTCAGGAAAT | CCAGCAGTGTGCTCAGGTTCAGTT |
| *Glut1* | GCAGATGATGCGGGAGAAGAAGG | GCCTTCTCGAAGATGCTCGTTGA |
| *Glut4* | CTGATTCTGCTGCCCTTCTGTCCT | GACATTGGACGCTCTCTCTCCAACTT |
| *Hsl* | CATGGCTCAACTCCTTCCTGGAAC | TTCAAGGTATCTGTGCCCAGTAAGCC |
| *Lpl* | CCAGGATGCAACATTGGAGAAGC | GCAGGGAGTCAATGAAGAGATGAATG |
| *Pgc-1a* | ACGTCCCTGCTCAGAGCTTCTCA | ATGTTGCGACTGCGGTTGTGTATG |
| *Ppara* | ACAAGTGCCTGTCTGTCGGGATG | TCCGAATCTTTCAGGTCGTGTTCA |
| *Pparg* | AGGGCGATCTTGACAGGAAAGACA | AAATTCGGATGGCCACCTCTTTGC |
| *Prdm16* | CACAGCACGGTGAAGCCATTCA | TAGTGCTGAACATCTGCCCACAGT |
| *Ucp1* | AGCCACCACAGAAAGCTTGTCAAC | ACAGCTTGGTACGCTTGGGTACTG |

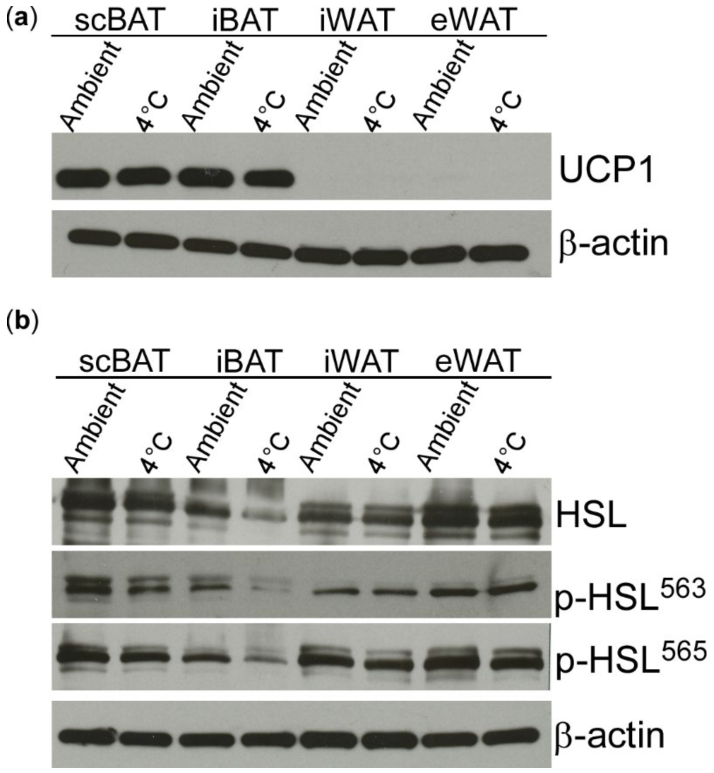


Figure S1. Protein levels of UCP1, HSL, and p-HSL in adipose tissues from mice housed at ambient temperature or after 6 hours of cold exposure. Representative images of western blot analysis of UCP1, HSL, and p-HSL (p-Ser$^{563}$ and p-Ser$^{565}$) in scBAT, iBAT, iWAT, and eWAT dissected from an 8-week-old mouse housed at ambient temperature or after 6 hours of cold exposure. (**a**) UCP1, (**b**) HSL, p-HSL$^{563}$, and p-HSL$^{565}$. β-actin was used as a loading control. Three independent western blot analyses were performed for UCP1, HSL, and p-HSL$^{565}$. Two independent western blot analyses were performed for p-HSL$^{563}$. p-HSL$^{565}$ has been shown to prevent phosphorylation of HSL at Ser$^{563}$ [60].

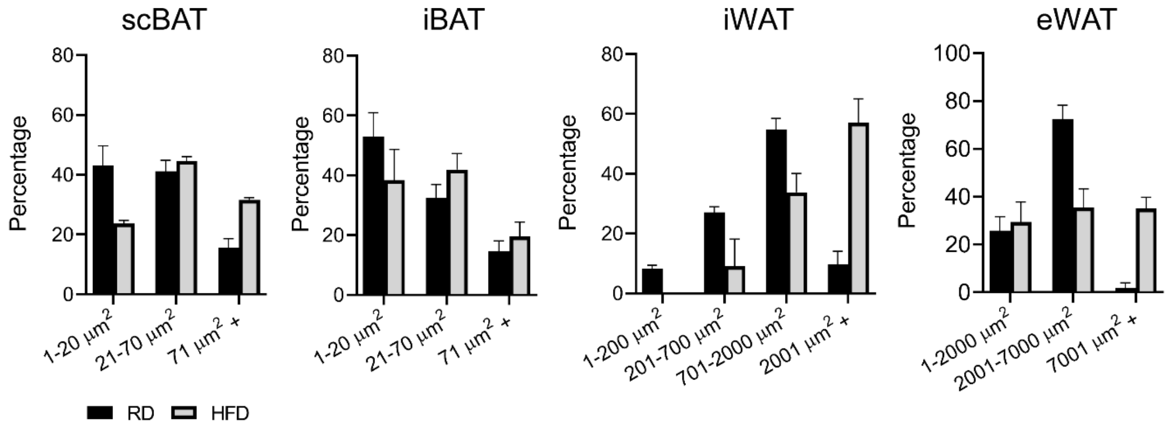


Figure S2. Size distributions for lipid droplets in scBAT, iBAT, iWAT, and eWAT from mice fed RD or 16 weeks of HFD. Lipid droplets were grouped based on their sizes (μm), small (BAT: 20 μm$^2$ or less, iWAT: 200 μm$^2$ or less, eWAT: 2000 μm$^2$ or less), medium (BAT: 21-70 μm$^2$, iWAT: 201-2000 μm$^2$, eWAT: 2001-7000 μm$^2$), or large (BAT: 71 μm$^2$ +, iWAT: 2001 μm$^2$ +, eWAT: 7001 μm$^2$ +). The distributions are presented as percentage of lipid droplets that are small, medium, or large within the total amount of lipid droplets counted. The percentage of lipid droplets in the large size group is higher in mice fed with HFD than with RD. Adipose tissue sections from 3 mice were counted.

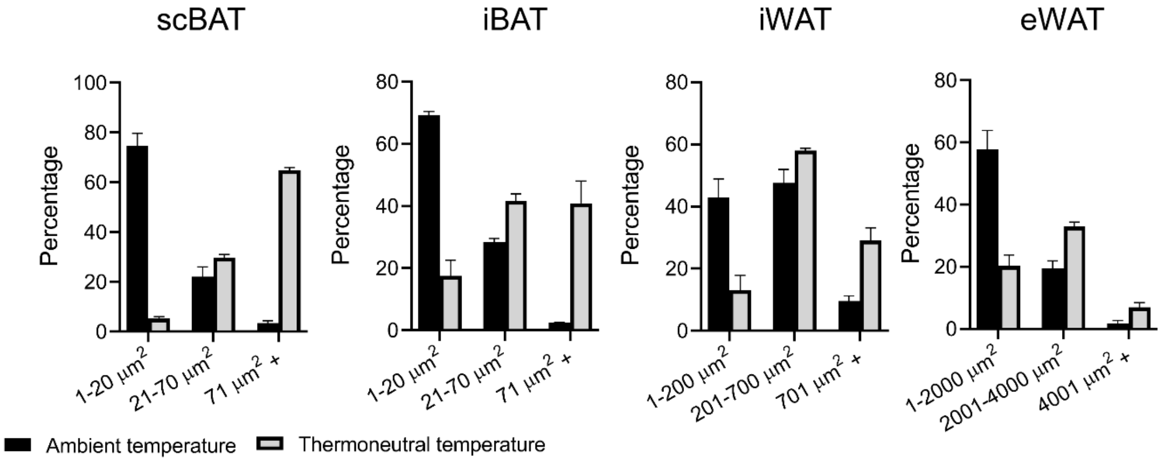


Figure S3. Size distributions for lipid droplets in scBAT, iBAT, iWAT, and eWAT from mice housed at ambient or thermoneutral temperature for 8 weeks. Lipid droplets were grouped based on their sizes (μm), small (BAT: 20 $\mu m^2$ or less, iWAT: 200 $\mu m^2$ or less, eWAT: 2000 $\mu m^2$ or less), medium (BAT: 21-70 $\mu m^2$, iWAT: 201-700 $\mu m^2$, eWAT: 2001-4000 $\mu m^2$), or large (BAT: 71 $\mu m^2$ +, iWAT: 701 $\mu m^2$ +, eWAT: 4001 $\mu m^2$ +). The distributions are presented as percentage of lipid droplets that are small, medium, or large among the total amount of lipid droplets counted. The percentage of lipid droplets in large size group is higher in mice housed in thermoneutral than in ambient temperatures. Adipose tissue sections from 3 mice were counted.

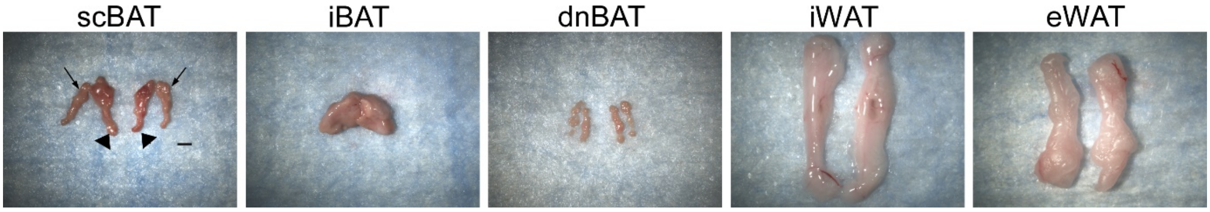


Figure S4. Adipose tissue images. Representative images of scBAT, iBAT, dnBAT, iWAT, and eWAT isolated from an 8-week-old male mouse. Scale bar: 1000 μm. Compared to scBAT and iBAT, dnBAT is much smaller. Arrowhead points to the part of scBAT that is positioned above the external jugular vein on the ventral side of the neck. Arrow points to the part of the scBAT that is positioned behind the external jugular vein.
